# A DISEASE SIMILARITY APPROACH IDENTIFIES A LINK BETWEEN ALZHEIMER’S DISEASE AND NIEMANN-PICK TYPE C DISEASE

**DOI:** 10.1093/geroni/igae098.2262

**Published:** 2024-12-31

**Authors:** Vikas Gujjala, Alaattin Kaya

**Affiliations:** Virginia Commonwealth University, Richmond, Virginia, United States; Virginia Commonwealth University, Richmond, Virginia, United States

## Abstract

Since its first description in 1906 by Dr. Alios Alzheimer, Alzheimer’s disease (AD) has been the most common type of dementia. Initially identified as caused by age-associated plaque accumulation, research has shifted to lysosomal storage and metabolic disorders, and pathogenesis from amyloid and tau accumulation to oxidative stress and impaired lipid and glucose metabolism aggravated by hypoxic conditions. However, the underlying mechanisms linking these processes to disease progression are undefined. Here, we applied a disease similarity approach on transcriptomic data of various congenital diseases with increased AD risk, namely Down Syndrome (DS), Niemann Pick Disease Type C (NPC), and Mucopolysaccharidosis I (MPS), to identify unknown molecular targets. We uncovered common pathways, hub genes, and miRNAs, many never associated with AD before. We discovered potential drug candidates for neuroprotection, and to ameliorate AD pathology through protein-drug interactions. Finally, we investigated molecular changes in brain samples from an NPC1 mouse model and analyzed the similarities to the brain samples of human and mouse AD models. We found a significant overlap between the NPC1 mice and AD human and mouse samples suggesting its potential in AD research as a comprehensive short-lived in vivo model. We believe our research represents the first comprehensive approach to congenital disease association with neurodegeneration and a new perspective on AD research while highlighting the lack of correlation in diverse in vitro models, which can accelerate diagnostic and therapeutic applications against dementia.

